# Novel immune checkpoint targets: moving beyond PD-1 and CTLA-4

**DOI:** 10.1186/s12943-019-1091-2

**Published:** 2019-11-06

**Authors:** Shuang Qin, Linping Xu, Ming Yi, Shengnan Yu, Kongming Wu, Suxia Luo

**Affiliations:** 1 Department of Oncology, Tongji Hospital of Tongji Medical College, Huazhong University of Science and Technology, 1095 Jiefang Avenue, Wuhan, 430030 Hubei China; 2 Department of Medical Oncology, The Affiliated Cancer Hospital of Zhengzhou University & Henan Cancer Hospital, Zhengzhou, 450008 China

**Keywords:** Immunotherapy, Immune checkpoint, LAG-3, TIM-3, TIGIT, VISTA, B7-H3, BTLA

## Abstract

The emergence of immune checkpoint inhibitors (ICIs), mainly including anti-programmed cell death protein 1/programmed cell death ligand 1 (PD-1/PD-L1) and anti-cytotoxic T lymphocyte-associated antigen-4 (CTLA-4) monoclonal antibodies (mAbs), has shaped therapeutic landscape of some type of cancers. Despite some ICIs have manifested compelling clinical effectiveness in certain tumor types, the majority of patients still showed de novo or adaptive resistance. At present, the overall efficiency of immune checkpoint therapy remains unsatisfactory. Exploring additional immune checkpoint molecules is a hot research topic. Recent studies have identified several new immune checkpoint targets, like lymphocyte activation gene-3 (LAG-3), T cell immunoglobulin and mucin-domain containing-3 (TIM-3), T cell immunoglobulin and ITIM domain (TIGIT), V-domain Ig suppressor of T cell activation (VISTA), and so on. The investigations about these molecules have generated promising results in preclinical studies and/or clinical trials. In this review, we discussed the structure and expression of these newly-characterized immune checkpoints molecules, presented the current progress and understanding of them. Moreover, we summarized the clinical data pertinent to these recent immune checkpoint molecules as well as their application prospects.

## Background

The past decade has witnessed the rapid development of immunotherapy. Now, it has been recognized as a key strategy to control the progression of malignant tumors. Among these immunotherapies, immune checkpoint inhibitors (ICIs) [1], chimeric antigen receptor T cell [2] and bispecific antibodies [3] are the most promising immunotherapy strategies. Encouragingly, the 2018 Nobel Prize in Physiology and Medicine was awarded to Drs. James Allison and Tasuku Honjo, who discovered programmed cell death protein-1 (PD-1) and cytotoxic T lymphocyte-associated antigen-4 (CTLA-4), to honor their outstanding work for the development of immunotherapy [4]. In addition, Dr. Lieping Chen did seminal contributions to the discovery of PD-L1 (CD274, B7-H1) [5]. Now, these immune checkpoint targets have realized the transformation from the laboratory to the clinical application.

CTLA-4 is a cell-surface receptor related to CD28, binding to the ligands CD80 (B7–1) and CD86 (B7–2) [6]. The binding of CTLA-4 to CD80/CD86 delivers a negative signal to T cells activation by making CD80/CD86 less available to CD28 [7]. In the early 1996, Leach and his colleagues found that injecting anti-CTLA-4 antibody in mice with pre-established tumors significantly reduced tumor growth [8]. Ipilimumab, as the first anti-CTLA-4 monoclonal antibody (mAb) reaching the clinic [9], garnered the approval for the treatment of patients with malignant melanoma in 2011 [10]. PD-1, also known as CD279, belongs to CD28 superfamily [11]. The binding of PD-1 to its ligands PD-L1 and PD-L2 (CD273, B7-DC) serves as a “rheostat” of immunological regulation, suppressing the activation and function of T cells to downregulate the immune response [12, 13]. Pembrolizumab, the first humanized mAb against PD-1, gained its first global approval for patients with unresectable or metastatic melanoma by United States Food and Drug Administration (FDA) in 2014 [14]. Subsequently, its indication was expended to head and neck squamous cell carcinoma (HNSCC) [15], non-small cell lung cancer (NSCLC) [16], metastatic urothelial carcinoma [17], cervical carcinoma [18] among others in a list that continues to grow. By the end of 2018, as many as 7 types of ICIs have been approved by FDA for the treatment of cancers and all of them were immune checkpoint blockers against PD-1/PD-L1 or CTLA-4 [19].

Nevertheless, the response rate of anti-PD-1/PD-L1 mAb and anti-CTLA-4 mAb in overall patients is far from satisfactory. Most patients show primary or acquired resistance to these ICIs [9]. Thus, intensive researches aimed at finding novel immune checkpoint targets have been ongoing. The next generation immune checkpoints such as lymphocyte activation gene-3 (LAG-3) [20], T cell immunoglobulin and mucin-domain containing-3 (TIM-3) [21], T cell immunoglobulin and ITIM domain (TIGIT) [22], V-domain Ig suppressor of T cell activation (VISTA) [23], B7 homolog 3 protein (B7-H3) [24] and B and T cell lymphocyte attenuator (BTLA) [25] demonstrate as promising therapeutic targets with the chance to realize clinical application. In this review, we will emphasize these newly-characterized immune checkpoint molecules and their clinical studies that suggest the promising future for the clinical application.

## Immune checkpoints

The full activation of T cell dependents on two different signals, signal one is derived from the interaction between antigenic peptide/major histocompatibility complex (MHC) on the surface of APCs and the T cell receptor (TCR), and signal two requires an antigen-independent co-signaling molecules [26]. Note worthily, T cell activation is tightly regulated by co-stimulators or co-inhibitors known as immune checkpoints [7]. If antigen/MHC and TCR binding is accompanied by the engagement of costimulatory receptors, such as CD28, it allows T cell to proliferate and to migrate toward specific antigen. On the contrary, if antigen/MHC and TCR binding is accompanied by the engagement of coinhibitory receptors, such as CTLA-4, it will suppress T cell activation [27, 28]. CTLA-4 is not detectable in naïve T cell but is rapidly induced upon T cell activation and it primarily regulates the amplitude of T cell during the early priming phase in lymphoid organs [29, 30]. The binding of CTLA-4 to B7 proteins competes CD28 costimulatory signals and eventually acts to impede excessive immunity [31]. The aim of this co-inhibitor is to minimize damage to normal tissues and prevent unwanted autoimmunity [31, 32]. In contrast to CTLA-4, PD-1 plays a major role in the maintenance of peripheral tolerance [33]. The engagement of PD-1 by its ligands results in the recruitment of Src homology 2 (SH2) domain containing phosphatases 1/2 (SHP1/2) and then inhibits T cell proliferation and cytokine secretion mediated by TCR [34]. Some cancer cells possess the ability to generate inhibitory ligands which can bind co-inhibitory receptor molecules. This engagement limits normal anti-tumor immune responses thus assisting in immune escape [35]. Therefore, the blockades of these immune checkpoints are capable to invoke patient’s own anti-tumor immune response [32]. Immune checkpoint therapies do not kill cancer cells directly, instead they harness the power of the host’s immune system to re-enhance endogenous anti-tumor activity [36].

## Newly emerging immune checkpoints

Apart from CTLA-4 and PD-1, novel immune checkpoint molecules on T cells have been discovered continuously. So far, all these emerging immune checkpoints targets are either in the clinical trial or under active development. Those delineated below are the most promising immune checkpoint targets for which blocking antibodies are available in clinical trials (Table 1). A substantial body of evidence accumulates to indicate the synergistic effect of combinatorial blockade among these new immune checkpoints and anti-PD-1/L1 and/or anti-CTLA-4 mAbs. Huang and his colleague utilized a murine model to explore the effect of combinatorial blockade of LAG-3 and PD-1 pathways in ovarian cancer [37]. Their results showed that dual blockade of LAG-3 and PD-1 synergistically enhanced anti-tumor immunity and suppressed tumor growth by enhancing CD8^+^ tumor infiltrating T cells (TILs) and decreasing regulatory T cells (Tregs) in the tumor microenvironment (TME) [37]. The same group further detected the level of other inhibitory receptors when PD-1 or LAG-3 was blocked. When the mice were treated with anti-PD-1 mAb, the level of LAG-3 and CTLA-4 were increased. Interesting, treatment with anti-LAG-3 mAb upregulated the level of PD-1 [38]. Their experiments indicated that the blockade of a single immune checkpoint targets may lead to compensatory upregulation of other checkpoint receptors in TME. The similar compensatory mechanism between TIM-3 and PD-1 was observed in lung cancer [39] and melanoma [40]. It seems that the compensatory mechanism is common across different types of cancer. These preclinical results pave the way for the combinatorial blockade strategies in clinical trials.

**Table 1:** The clinical trials of novel immune checkpoint inhibitors in cancer immunotherapy

Target	Drugs (company)	Combination agents	Phase	Tumor types	Clinical Trial NO.	State
LAG-3	IMP321/Eftilagimod alpha (Immutep)	–	I	Metastatic RCC	NCT00351949	Completed
		Paclitaxel	I	MBC	NCT00349934	Completed
		Cyclophosphamide, fludarabine, Melan-A VLP vaccine	I	Metastatic melanoma	NCT00324623	Completed
		HLA-A2 peptides	I/II	Disease-free melanoma	NCT00365937	Terminated
		Gemcitabine	I	Advanced pancreas cancer	NCT00732082	Terminated
		Tumor antigenic peptides, montanide	I/II	Advanced melanoma	NCT01308294	Terminated
		Paclitaxel	II	Metastatic breast cancer	NCT02614833	Active, not recruiting
		Pembrolizumab	I	Metastatic melanoma	NCT02676869	Active, not recruiting
		–		Advanced solid tumors	NCT03252938	Recruiting
		Pembrolizumab	II	Advanced NSCLC and HNSCC	NCT03625323	Recruiting
	Relatlimab /BMS-986016 (BMS)	Nivolumab	I/II	Advanced solid tumors	NCT01968109	Recruiting
		Nivolumab	I	Advanced solid Tumors	NCT02966548	Recruiting
		Nivolumab and Urelumab	I	Recurrent glioblastoma	NCT02658981	Recruiting
		Nivolumab	I	Recurrent glioblastoma	NCT03493932	Recruiting
		Nivolumab, Carboplatin, Paclitaxel, Radiation	I	Gastro/esophageal cancer	NCT03044613	Recruiting
		Nivolumab, Cabiralizumab, Ipilimumab, anti-GITR, IDO1 Inhibitor, Lirilumab, Radiation	I	Advanced solid tumors	NCT03335540	Recruiting
		Nivolumab, Ipilimumab	I/II	Virus-associated tumors	NCT02488759	Recruiting
		Nivolumab	I/II	Advanced hematologic malignancies	NCT02061761	Recruiting
		Nivolumab, Ipilimumab, BMS-986205	I/II	Advanced solid tumors	NCT03459222	Recruiting
		Nivolumab	II	Advanced chordoma	NCT03623854	Recruiting
		Nivolumab	II	Metastatic melanoma	NCT03743766	Recruiting
		Nivolumab	II	MSS advanced CRC	NCT03642067	Recruiting
		Nivolumab	II	MSI-H solid tumors	NCT03607890	Recruiting
		Nivolumab, Ipilimumab, BMS-986205, BMS-813160	II	Advanced RCC	NCT02996110	Recruiting
		Nivolumab, Ipilimumab, BMS-986205	II	Advanced GC	NCT02935634	Recruiting
		Nivolumab, Dasatinib, Ipilimumab, BMS- 986205	II	Advanced NSCLC	NCT02750514	Active, not recruiting
		Ipilimumab, Nivolumab, Cobimetinib, Daratumumab, anti-LAG-3 antibody	II	Advanced CRC	NCT02060188	Active, not recruiting
		Nivolumab, Ipilimumab	II	Melanoma	NCT02519322	Recruiting
	LAG525 (Novartis)	PDR001	I/II	Advanced solid tumors	NCT02460224	Active, not recruiting
		PDR001, NIR178, capmatinib, MCS110, canakinumab	I	TNBC	NCT03742349	Recruiting
		PDR001	II	Advanced solid and hematologic malignancies	NCT03365791	Active, not recruiting
		PDR001, carboplatin	II	Advanced TNBC	NCT03499899	Recruiting
		PDR001, capmatinib, canakinumab, ribociclib	II	Advanced melanoma	NCT03484923	Recruiting
	MK-4280 (Merck)	Pembrolizumab, Oxaliplatin, Irinotecan, Leucovorin, 5-FU, MK-4280A	I	Advanced solid tumors	NCT02720068	Recruiting
		pembrolizumab	I/II	Hematological malignancies	NCT03598608	Recruiting
		Pembrolizumab, Lenvatinib, MK-1308	II	Advanced NSCLC	NCT03516981	Recruiting
	REGN3767 (Regeneron)	REGN2810	I	Advanced Cancers	NCT03005782	Recruiting
	TSR-033 (Tesaro)	Anti-PD-1	I	Advanced solid tumors	NCT03250832	Recruiting
	BI754111 (Bohringer Ingelheim)	BI754091	Early I	Neoplasms	NCT03433898	Recruiting
		BI754091	I	Advanced cancers	NCT03156114	Recruiting
		BI754091	I	Advanced NSCLC and HNSCC	NCT03780725	Recruiting
		BI754091	II	Advanced solid tumors.	NCT03697304	Recruiting
		BI754091, BI907828	I	Advanced solid tumors.	NCT03964233	Recruiting
	Sym022 (Symphogen)	–	I	Advanced solid tumor or lymphomas	NCT03489369	Recruiting
		Sym021, Sym023	I	Advanced solid tumor or lymphomas	NCT03311412	Recruiting
	FS118^**a**^ (F-star)	–	I	Advanced malignancies	NCT03440437	Recruiting
	MGD013^**b**^ (MacroGenics)	–	I	Advanced cancers	NCT03219268	Recruiting
TIM-3	TSR-022 (Tesaro)	TSR-042, TSR-033	I	Advanced solid tumors	NCT02817633	Recruiting
		Niraparib, TSR-042, Bevacizumab, Platinum-Based chemotherapy	I	Advanced solid tumors	NCT03307785	Recruiting
		TSR-042	II	Liver Cancer	NCT03680508	Not yet recruiting
	MBG453 (Novartis)	PDR001	I/II	Advanced malignancies.	NCT02608268	Recruiting
		Decitabine, PDR001	I	AML or high risk MDS	NCT03066648	Recruiting
		HDM201, Venetoclax	I	AML or high risk MDS	NCT03940352	Recruiting
		Spartalizumab	I	GBM	NCT03961971	Not yet recruiting
	Sym023 (Symphogen)	–	I	Advanced solid tumor or lymphomas	NCT03489343	Recruiting
		Sym021, Sym022	I	Advanced solid tumor or lymphomas	NCT03311412	Recruiting
	INCAGN2390 (Incyte)	–	I	Advanced malignancies	NCT03652077	Recruiting
	LY3321367 (Eli Lilly and Company)	LY3300054	I	Advanced solid tumor	NCT03099109	Recruiting
		LY3300054, Ramucirumab, Abemaciclib, Merestinib	I	Advanced solid tumor	NCT02791334	Recruiting
	BMS-986258 (BMS)	Nivolumab, rHuPH20	I/II	Advanced solid tumor	NCT03446040	Recruiting
	SHR-1702 (Jiangsu HengRui)	Camrelizumab	I	Advanced solid tumor	NCT03871855	Not yet recruiting
	RO7121661^c^ (Roche)	–	I	Advanced solid tumor	NCT03708328	Recruiting
TIGIT	MK-7684 (Merck)	Pembrolizumab	I	Advanced solid tumor	NCT02964013	Recruiting
	Etigilimab /OMP-313 M32 (OncoMed)	Nivolumab	I	Advanced solid tumor	NCT03119428	Active, not recruiting
	Tiragolumab/MTIG7192A/RG-6058 (Genentech)	Atezolizumab	I	Advanced solid tumor	NCT02794571	Active, not recruiting
		Atezolizumab	II	Advanced NSCLC	NCT03563716	Active, not recruiting
	BMS-986207 (BMS)	Nivolumab	I/II	Advanced solid tumor	NCT02913313	Recruiting
	AB-154 (Arcus Biosciences)	AB122	I	Advanced malignancies	NCT03628677	Recruiting
	ASP-8374 (Potenza)	Pembrolizumab	I	Advanced solid tumors	NCT03260322	Recruiting
		–	I	Advanced solid tumor	NCT03945253	Not yet recruiting
VISTA	JNJ-61610588 (Johnson & Johnson)	–	I	Advanced solid tumor	NCT02671955	Terminated
	CA-170^d^ (Curis)	–	I	Advanced solid tumors and lymphomas	NCT02812875	Active, not recruiting
B7-H3	Enoblituzumab /MGA271 (MacroGenics)	–	I	Advanced solid tumors	NCT01391143	Active, not recruiting
		Ipilimumab	I	Advanced solid tumors	NCT02381314	Completed
		Pembrolizumab	I	Advanced solid tumors	NCT02475213	Active, not recruiting
		–	I	Children with B7-H3-expressing solid tumors	NCT02982941	Completed
		–	II	Prostate cancer	NCT02923180	Recruiting
	MGD009^e^ (MacroGenics)	MGA012	I	Advanced solid tumors	NCT03406949	Recruiting
		–	I	B7-H3-expressing tumors	NCT02628535	Recruiting
	^131^I-8H9 /omburtamab (Y-mAbs)	–	I	DSRCT	NCT01099644	Recruiting
		–	I	Advanced CNS or leptomeningeal cancer	NCT00089245	Recruiting
		–	II/III	Neuroblastoma central nervous system/leptomeningeal metastases	NCT03275402	Recruiting
	^124^I-8H9 /omburtamab (Y-mAbs)	–	I	Gliomas	NCT01502917	Recruiting

Abbreviation: ^a^, a bispecific anti-LAG-3/PD-L1 antagonistic mAb; ^b^, a bispecific anti-LAG-3/PD-1 antagonistic mAb; ^c^, a bispecific anti-TIM-3/PD-1 antagonistic mAb; ^d^, an oral inhibitor targeted PD-L1 and VISTA; ^e^, a bispecific mAb designed to bind CD3 on T cells and B7-H3 on tumor; *BMS* Bristol-Myers Squibb, *RCC* Renal cell carcinoma, *MBC* Metastatic breast cancer, *NSCLC* Non-small cell lung cancer, *HNSCC* Squamous cell carcinoma of the head and neck, *CRC* Colorectal cancer, *TNBC* Triple Negative Breast Cancer, *AML* Acute Myeloid Leukemia, *MDS* Myelodysplastic, *MSS* Microsatellite stable, *MSI-H* Microsatellite instability high, *GC* Gastric Cancer, *DSRCT* Desmoplastic Small Round Cell Tumors, *CNS* Central nervous system, *GBM* Glioblastoma multiforme

### Lymphocyte activation Gene-3 (LAG-3)

#### The biology of LAG-3

**Fig. 1:**
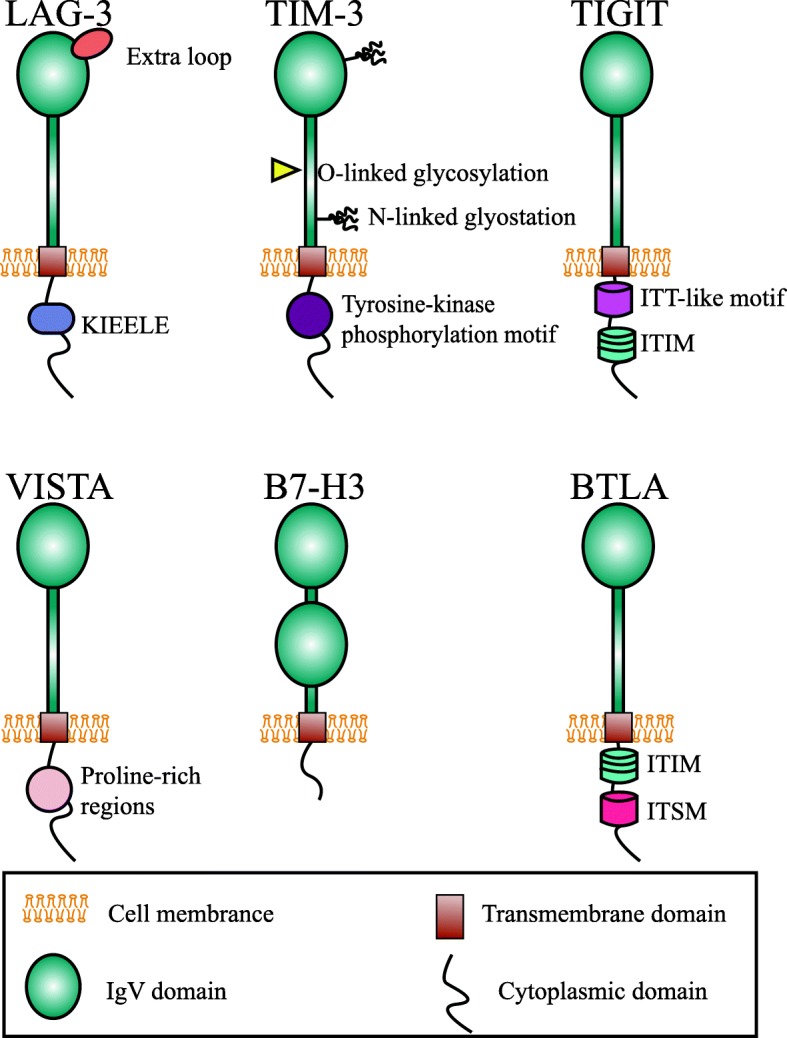
Structure of LAG-3, TIM-3, TIGIT, VISTA, B7-H3, and BTLA. All of them were type I transmembrane glycoprotein with a similar structure, including N-terminal IgV domain, a transmembrane domain and a cytoplasmic tail. However, they share distinct signaling motif

LAG-3 (CD223) was discovered by Triebel and colleagues as early as 1990 [41]. The LAG-3 gene encompass 8 exons and the corresponding cDNA can encode a 498-amino acid type I membrane protein [41]. LAG-3 gene is located adjacent to CD4 gene on chromosome 12, and further analysis of amino acid sequence reveals an approximately 20% identical to CD4 (Table 2) [41]. Mature LAG-3 protein includes four parts, hydrophobic leader, extracellular region, transmembrane region, and cytoplasmic region (Fig.1). The extracellular region is consisted of four immunoglobulin (Ig) superfamily-like domains (D1-D4) [42]. The membrane-distal D1 domain contains a unique short amino acid sequence, the so-called “extra loop” [43]. The cytoplasmic domain of LAG-3 has three conserved regions: a serine-phosphorylation site, a KIEELE motif, and a glutamic acid-proline repeats, of which the KIEELE motif is essential for LAG-3 to exert inhibitory function [44]. Metalloproteases can cleave LAG-3 within the connecting peptide between the D4 transmembrane domain and the transmembrane domain, generating a soluble LAG-3 (sLAG-3) [45]. Some researches demonstrated that sLAG-3 could limit the magnitude of the T cell immune responses [46]. LAG-3 is usually expressed on activated CD4^+^ and CD8^+^ T cells [41], Tregs [47], a subpopulation natural killer (NK) cells [48], B cells [49], plasmacytoid dendritic cells (pDCs) as well [50]. Ample of evidence have indicated that LAG-3 signaling play a negative regulatory role in T helper 1 (Th1) cell activation, proliferation and cytokine secretion [51–53]. During tumorigenesis and cancer progression, tumor cells exploit this pathway to escape from immune surveillance.

**Table 2:** Comparison of coinhibitory immune checkpoint receptors mentioned in manuscript

Receptor	LAG-3	TIM-3	TIGIT	VISTA	B7-H3	BTLA
Alternate name	CD223	HAVCR2	WUCAM/ Vstm3/ Vsig9	PD-1H/DD1α/ Gi24/Dies1/B7-H5	CD276	CD272
Chromosomal location	12p13.32	5q33.2	3q13.31	10q22.1	15q24.1	3q13.2
Function of ligand-receptor interaction	Co-inhibition	Co-inhibition	Co-inhibition	Co-inhibition	Co-inhibition or co-stimulation	Co-inhibition
Binding Partner	MHC-II, galectin-3, LSECtin, a-synuclein, FGL1	Galectin-9, Ceacam-1, HMGB-1, PtdSer	CD155, CD112	VSIG-3	Unknow	HVEM
Number of amino acids	498 amino acids	302 amino acids	244 amino acids	311 amino acids	316 amino acids	289 amino acids
Signaling motif	KIEELE motif	Tyrosine residues	ITT and ITIM	Unknow	Unknow	ITIM and ITSM
Receptor Expression	Activated T cells, B cells, Tregs, NK cells, DCs	Activated T cells, B cells, Tregs, DCs, NK cells, monocytes	T cells, NK cells	Myeloid cells, T cells	Activated T cells, NK cells, DCs, monocytes, tumor tissue	Mature B cells, T cells, Tregs, macrophages, DCs

**Fig. 2:**
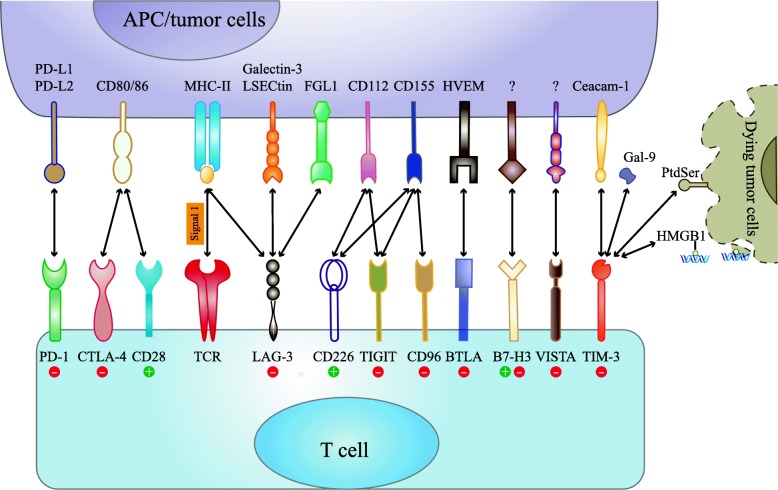
Current and emerging immune checkpoint receptors and their respective ligands. Various immune checkpoint molecules expressed on T cells were shown with their ligands. Immune checkpoints such as PD-1, CTLA-4, LAG-3, TIM-3, TIGIT bound with their respective ligands on APCs and/or tumor cells, triggering a negative or positive signal to T cells response

Based on the structural similarity between LAG-3 and CD4, MHC-II is reasonable considered as ligand for LAG-3. In fact, the binding affinity between LAG-3 and MHC-II is 100-fold higher than CD4 [20]. Now, MHC-II [54], galectin-3 [55], LSECtin [56], and a-synuclein [57] have been described to interact with LAG-3, with the MHC-II as a canonical ligand (Fig. 2). More recently, Wang and his co-workers elucidated that fibrinogen-like protein 1 (FGL1) was a novel high-affinity ligand for LAG-3 independent from MHC-II [58].

#### Clinical trials on LAG-3

Based on the experimental results that administration of recombinant sLAG-3 molecule with irradiated tumor cells can dampen the growth of established tumors, early clinical work centered on developing a sLAG-3-Ig [59]. IMP321 (Eftilagimod alpha) was initially developed as a vaccine immunological adjuvant [60]. It is a soluble recombinant protein by fusing the four extracellular Ig domains of LAG-3 to the Fc portion of human IgG1 [61]. The first-in-man phase I trial was conducted in patients with metastatic renal cell carcinoma (mRCC) (NCT00351949) [62]. The results showed that adverse events (AEs) related to IMP321 were minimal and IMP321 increased the subset of circulating activated CD8^+^ T cells which was correlated to tumor growth reduction. Although no objective response was observed in this trial, 7 of 8 patients experienced stable disease in higher IMP321 dose group (> 6 mg) while only 3 of 11 in the lower dose group [62]. IMP321 monotherapy showed a modest efficacy in cancer therapy, thus it was rational to combine the agent with other chemotherapeutic drugs. Subsequently, another two clinical trials were lunched in metastatic breast cancer (MBC) (NCT00349934) [63] and advanced pancreatic cancer (NCT00732082) [64]. Ultimately, the former phase I/II trial showed a 50% objective response rate (ORR) at the end of treatment point, which compared favorably to a historical response rate of approximately 25% [63]. Furthermore, the authors found an absolute and proportional increase in MHC class II-expressing APCs, NK cells and CD8^+^ T cell populations and these subsets were known to connect with antitumor activity [63]. This encouraging result has prompted a further phase IIb multicenter clinical trial that is currently recruiting patients with MBC (NCT02614833) [65]. Unfortunately, the later phase I clinical trial intended to assess the role of IMP321 and gemcitabine as a front-line therapy in patients with pancreatic cancer showed no meaningful objective response [64]. The role of IMP321 in combination with other immunotherapies (e.g., anti-PD-1 mAb, NCT03625323) or as an adjuvant for cancer vaccines are being explored (NCT00324623, NCT01308294) [66, 67].

Relatlimab, also named BMS-986016, is the first commercially mAb directed against LAG-3 [68]. Many preclinical mouse models have showed that PD-1/PD-L1 blockade upregulated LAG-3 or other immune checkpoints as a compensatory mechanism [37, 38, 69]. These data evoked the further exploration of combination therapy strategies. For example, the first phase I clinical trial about Relatlimab was opened in 2013 (NCT01968109) [38]. The aim of this clinical trial was to evaluate the efficacy of Relatlimab as a monotherapy or in combination with Nivolumab (anti-PD-1 antibody) in patients with various advanced malignancies including melanoma, NSCLC, and RCC [20]. At ESMO 2017, researcher announced the updated efficacy and safety results in a cohort of 68 melanoma patients who had received prior immunotherapy [70]. The ORR was 11.5% in 61 patients who were able to assess efficacy, including one patient achieved complete response and 6 were partial response (PR). Noticeably, the ORR was higher in patients with the expression of LAG-3 ≥ 1% and the AEs were tolerable [70]. As of July 15, 2019, at least 18 clinical trials on Relatlimab had been registered on the ClinicalTrials.gov. All of them were phase I or II but none of them are completed.

LAG525 is another humanized anti-LAG-3 mAb. It is a high-affinity IgG4 antibody which blocks the binding of MHC-II to LAG-3. Currently, LAG525 is undergoing a series of phase I or II testing in combination with anti-PD-1 antibody for patients with advanced cancers. For example, LAG525’s first clinical trial was launched in 2015 (NCT02460224). It was a phase I trial to determine the efficacy and safety of LAG525 plus PDR001 (anti-PD-1 mAb) in advanced malignancies [36]. Other LAG-3 inhibitory antibodies MK-4280, REGN3767 [71], TSR-033 [72], BI754111 [73], and Sym022 [74] have also been investigated at various stages of clinical development. To capitalize on synergistic effects of co-blockade PD-1 and LAG-3 pathways in preclinical models [75], some bispecific anti-LAG-3/PD-(L)1 antagonistic mAbs have also been developed, such as FS118 [76] and MGD013 [77]. To date, at least 10 kinds of LAG-3 blockade agents have been developed and studied in clinical trials, yet their results are not available now.

### T cell immunoglobulin and mucin-domain containing-3 (TIM-3)

#### The biology of TIM-3

TIM-3, also called hepatitis A virus cellular receptor 2 (HAVCR2), presents several unique features making it another intriguing immune checkpoint [78]. It was first identified as a protein selectively expressed on CD4^+^ Th1 and CD8^+^ T cytotoxic 1 (Tc1) cells as early as 2002 [21]. But now it is commonly classified as immune checkpoint molecule similar to CTLA-4 and PD-1. The genomic analysis shows that the TIM gene family is composed of three genes, namely TIM-1, TIM-3, TIM-4, located on human chromosome 5q33.2 [79]. Human TIM-3 protein comprises of 302 amino acids, while mouse homolog includes 281 amino acids residues with 63% identity to human TIM-3 [21]. It belongs to Ig superfamily (IgSF) with an N-terminal Ig variable region (IgV)-like domain, a membrane-proximal mucin-like domain containing sites for O-linked glycosylation (glycosylated mucin domain), a single transmembrane region and a C-terminal cytoplasmic tail. There are also sites for N-linked glycosylation between the mucin and transmembrane [79]. The TIM-3 cytoplasmic tail does not have the classical inhibitory signaling motif, like immune receptor tyrosine based inhibitory motif (ITIM) or immune receptor tyrosine-based switch motif (ITSM), but contains five conserved tyrosine residues, two of which (Y265 and 272) can be phosphorylated by Src kinases or interleukin inducible T cell kinase and are crucial for downstream signaling [35, 80]. The expression of TIM-3 was not limited on T cell, it was known to express on different types of immune cells, including B cells, Tregs, NK cells, DCs, monocytes, and macrophages [81]. Lately, the expression of TIM-3 has been identified in leukemic stem cells and tumor-associated endothelium [82, 83].

Hitherto, four distinct ligands have been reported to bind to the IgV domain of TIM-3, including galectin-9, high-mobility group protein B1 (HMGB1), carcinoembryonic antigen cell adhesion molecule 1 (Ceacam-1), and phosphatidyl serine (PtdSer) [84]. It is noteworthy that galectin-9 and HMGB1are soluble ligands, while Ceacam-1 and PtdSer belong to surface ligands. The engagement of TIM-3 with galectin-9 triggered intracellular calcium flux of Th1 cells, inducing cell death [85]. Furthermore, a study by Kang et al. showed that galectin-9 also induced apoptosis of TIM-3^+^CD8^+^T cell in colon cancer [86]. The interaction between HMGB1 and TIM-3 mainly had an impact on innate immune response. In tumor, TIM-3 was highly expressed on tumor infiltrating DCs and can compete with nucleic acid binding to HMGB1, therefore dampening anti-tumor immunity mediated by nucleic acids [87]. Ceacam-1 was a molecule involved in T cell inhibition. Huang and his colleague elucidated that TIM-3 and Ceacam-1 can form a heterodimer in both *cis* and *trans* which acts as a negative regulator of T cell responses [88]. The interaction of PtdSer with TIM-3 has been showed to connect with the clearance of apoptotic bodies and also improve the antigen cross-presentation [89]. More importantly, higher expression of TIM-3 was associated with a poor prognosis in solid malignant [90] and accumulating preclinical models have verified the therapeutic benefit of TIM-3 blockade by regulating TME and restricting tumor growth especially in combination with PD-1 blockade [91].

#### Clinical trials on TIM-3

To date, at least eight TIM-3 antagonistic mAbs have been registered on ClinicalTrials.gov. TSR-022 (Cobolimab), a novel IgG4 anti-TIM-3 mAb developed by Tesaro entered the first phase I clinical trial in 2016 (NCT02817633) [92]. This multicenter, open-label study intended to evaluate the safety and efficacy of TSR-022 as a monotherapy or in combination with TSR-042 (anti-PD-1 mAb) in patients with advanced solid tumor. The results have been released in 2018 Annual Meeting of the Society for Immunotherapy of Cancer (SITC) Conference [93]. Clinical benefits have been observed in the combination group, especially at a high dose of TSR-022 (300 mg) with a 15% ORR (3/20) and 40% stable disease (8/20) [93]. Another two clinical trials including TSR-022 are still recruiting patients with no clinical results available (NCT03307785, NCT03680508). MBG453 is another anti-TIM-3 mAb produced by Novartis. Similar to TSR-022, the first clinical trial aimed to assess the safety and efficacy of MBG453 as single agent or in combination with PDR001 (an anti-PD-1 mAb) in advanced malignancies patients (NCT02608268). Another clinical trial was conducted in patients with acute myelocytic leukemia or high-risk myelodysplastic syndromes (NCT03066648). Sym023, is a recombinant, fully human antibody that bound TIM-3 [94]. A phase I trial evaluating the safety, tolerability, and dose-limiting toxicities of sym023 is recruiting at present (NCT03489343). Other TIM-3 inhibitors INCAGN2390, LY3321367, BMS-986258 and SHR1702 are also being tested in phase I trial alone (INCAGN02390 NCT03652077) or in combination with anti-PD-1/PD-L1 mAb (LY3321367 NCT03099109; BMS-986258 NCT03446040; SHR1702 NCT03871855) in the metastatic setting [84, 95]. RO7121661 is a bispecific antibody targeting PD-1 and TIM-3 simultaneously. It was developed by Roche and a phase I dose escalation and expansion study has been ongoing on advanced solid tumors (NCT03708328).

### T cell immunoglobulin and ITIM domain (TIGIT)

#### The biology of TIGIT

TIGIT was first identified by Yu and his colleagues as an immune checkpoint rheostat that suppress the activation of T cells in 2009 [22]. Subsequently, it was described by other groups with each group giving a different name including WUCAM [96], Vstm3 [97], and Vsig9 [98]. TIGIT gene is located on human chromosome 3q13.31 and encodes a 244-amino acid transmembrane glycoprotein. The protein includes an extracellular IgV region, a transmembrane domain, and a cytoplasmic tail that harbors a canonical ITIM and an immunoglobulin tail tyrosine (ITT)-like phosphorylation motif [22]. The expression of TIGIT was demonstrated to be tightly restricted to lymphocytes, mainly on T cell subsets (including Tregs and memory T cells) and NK cells [22, 99]. TIGIT binds two ligands, namely CD155 (PVR or Necl-5) and CD112 (nectin-2, also known as PRR2 or PVRL2) with different affinity. Whether nectin-3 is another ligand for TIGIT is still in question [99]. TIGIT exerts its immunosuppressive effects by competing with other counterparts, CD266 (DNAM-1) or CD96 [100]. CD226 delivered a positive co-stimulatory signal, while TIGIT delivered inhibitory signals. This group of interacting proteins formed a co-stimulatory axis that are similar to the CTLA-4/B7/CD28 pathway [101].

As TIGIT was initially identified by a genomic search for structures shared a conserved ITIM motif, its immunosuppressive effects were delineated as expected. The initial research believed that TIGIT suppressed T cell activation in an indirect way. Specifically, the engagement of TIGIT with CD155 on DCs induced phosphorylation of CD155 and Erk, increased the secretion of IL-10, thus inhibiting T cell responses indirectly [22]. Subsequent studies demonstrated that TIGIT could also directly suppress T cell function by competing with CD226 [97, 102]. The role of TIGIT molecule in NK cells has been well studied. Stanietsky et al. indicated that ligation of TIGIT could lead to the inhibition of NK cells cytotoxicity through its cytoplasmic ITIM domain both in human and mouse [99, 103]. Furthermore, the major role of ITT-like motif in negatively modulating NK cells has been proved by two independent studies [104, 105]. Work from the Kurtulus group showed that the expression of TIGIT on Tregs was critically involved in Treg suppressive function [106]. Interestingly, Gur and his co-works discovered that TIGIT could directly bind to the Fap2 protein derived from Fusobacterium nucleatum, triggering a negative signal to suppress the activities of NK cells and T cells, and hence mediating a tumor-immune evasion mechanism [107]. Many groups generated agonistic anti-TIGIT mAb to verify the effect of TIGIT, indeed, they consistently reported a direct inhibitory effect on T cell proliferation [97, 102, 108]. Recently, some groups have reported that co-blockade of TIGIT with other checkpoint receptors, such as PD-1 and TIM-3, can exert synergistic effects in regulating antitumor responses [106, 109, 110].

#### Clinical trials on TIGIT

Based on the promising preclinical results, targeting TIGIT as a strategy for cancer treatment attracts the attention of many pharmaceutical companies, especially combined with ant-PD-1/PD-L1 mAb. There are at least six major agents targeting TIGIT now, focusing on three products. MK-7684, a candidate anti-TIGIT drug developed by Merck entered into a phase I clinical trial to analysis the safety, efficacy, and pharmacokinetics of MK-7684 as monotherapy and in combination with pembrolizumab in metastatic solid tumors (NCT02964013) [111]. The early phase I data was announced at the SITC’s 3rd Annual Meeting in 2018. Sixty eight individuals were enrolled with 34 patients in the monotherapy and 34 patients in combination arms. Finally, one PR and eight PR were observed in these two groups, and the disease control rates were 35 and 47%, respectively [112]. Etigilimab (OMP-313 M32) is a humanized mAb that developed to block TIGIT from binding CD155. It was developed by OncoMed/Celgene and entered the first phase I clinical trial in April 2017 (NCT03119428). This open-label research was designed to evaluate the safety and tolerability of Etigilimab as a single agent or in combination with an anti-PD-1 mAb in patients with advanced malignancies. At the 2017 American Association for Cancer Research Annual Meeting, OncoMed presented some positive results from several preclinical trials, thus its clinic performance was worth pursuing [111]. Another anti-TIGIT candidate drugs made by Genentech was named Tiragolumab (MTIG7192A, RG-6058). It’s also a fully human mAb designed to engage to TIGIT and hinder its interaction with CD155. There were two clinical trials about Tiragolumab registered on ClinicalTrials. gov (NCT02794571, NCT03563716). Other drugs, such as BMS-986207 made by Bristol-Myers Squibb, AB-154 made by Arcus biosciences and ASP-8374 made by Potenza also have initiated their phase I clinical trial with no clinical results reported [111].

### VISTA, B7-H3, BTLA, and Siglec-15

Apart from these three new immune checkpoints mentioned above, many other immune checkpoint co-inhibitors are also attractive targets, with a few drugs step into clinical trials. VISTA, is also known as PD-1 homolog (PD-1H), DD1α, Gi24, differentiation of embryonic stem cells 1 (Dies1), and B7-H5 [113]. It was first described as an IgSF ligand which can negatively regulate T cell responses in mouse [23]. Subsequently, the same laboratory presented the characteristic of human VISTA [114]. It is a type I transmembrane protein with an extracellular IgV domain, a stalk region, a transmembrane segment, and a cytoplasmic tail. Structural analysis shows that the IgV domain of VISTA shares a sequence homology both to CD28 and B7 families, while the full-length VISTA harbors a highest identity with PD-1 [115]. But unlike PD-1, VISTA don’t include a classical ITIM or ITSM motif in the cytoplasmic domain, the intracellular tail contains two potential protein kinase C binding sites and a proline rich motif that may function as docking sites, suggesting that VISTA has the potentially function as both a receptor and a ligand [115]. Up to now the counter structures for VISTA has not been well identified [116], and VSIG-3 was reported as a novel ligand for VISTA a short time ago [117]. VISTA was highly expressed on myeloid cells and a lesser extent on T cells, but not on tumor cells within the TME [118]. The preclinical studies on multiple murine models showed that VISTA blockade improved the infiltration, proliferation, and effector function of tumor-infiltrating T cells within the TME, thus altered the suppressive character of the TME [118]. JNJ-61610588 is a fully human IgG1 anti-VISTA mAb made by Johnson & Johnson. The phase I clinical trial was intended to evaluate safety and pharmacokinetics of JNJ-61610588 in patients with advanced cancers (NCT02671955) [119]. Another candidate, CA-170, is an oral inhibitor which can selectively target both PD-L1 and VISTA. The results from preclinical models showed remarkable anti-tumor effects with well-tolerance and the phase I clinical trial in patients with advanced solid tumor and lymphomas is currently recruiting (NCT02812875) [119]. Based on the newest result published by Blando et al., VISTA was regard as a promising target for patients with pancreatic cancer [120] and the HMBD-002, a novel anti-VISTA antibody developed by Hummingbird Bioscience, have received a financial assistance from Cancer Prevention and Research Institute of Texas (CPRIT) with the plan to initiate clinical trials in 2020.

B7-H3, also named CD276, is a type I transmembrane glycoprotein that is encoded on human chromosome 15 [24]. It was discovered as early as 2001 [24]. The initial study described it as a positive co-stimulator for it can stimulate the T cell response and IFN-γ production [24]. But recently studies reported that B7-H3 was involved in the inhibition of T cells [121, 122]. The receptor for B7-H3 has not yet been identified and it may explain the intricate immunomodulatory activity of B7-H3 for it may have more than one binding partner with distinct function [123]. The expression of B7-H3 protein can be detected on activated immune cells such as T cells, NK cells, and APCs. More importantly, it was overexpressed on a wide spectrum of tumor tissue and linked to disease states and prognosis [124]. Recently, Enoblituzumab (MGA271), an engineered Fc humanized IgG1 mAb against B7-H3, has been developed and brought to clinic trials [125]. Among the five clinical trials about Enoblituzumab, one of which had been completed but did not reported the final results. Another agents MGD009, is a bispecific mAb designed to bind both CD3 on T cells and B7-H3 on tumor cells [36]. It is being studied on two phase I clinical studies in patients with B7-H3 expression (NCT02628535, NCT03406949) [36]. Furthermore, 8H9 (omburtamab) is an antibody specific to B7-H3 [126]. It has showed a positive clinical efficacy as an antibody drug conjugate after it was labeled with radioactive iodine-131 (^131^I) and administrated to patients with metastatic central nervous system (CNS) neuroblastoma [127]. Currently, clinical trials with radiolabeled 8H9 have been evaluated on peritoneal cancers, gliomas, and CNS (NCT01099644, NCT01502917, NCT00089245 et al.) The newest result of NCT01502917 supported the further study in expanded cohort [128].

BTLA (CD272) is identified as another inhibitory receptor that belongs to CD28 superfamily [113]. It is located on human chromosome 3 in q13.2 and encodes a 289-amino acid type I glycosylated transmembrane protein [25]. Similar to PD-1 and CTLA-4, the protein structure of BTLA includes a single extracellular region, a transmembrane domain and cytoplasmic domain. The ITIM and ITSM within the cytoplasmic tail mediate a negative signaling to T cells by recruiting the SHP-1 and SHP-2 [129]. BTLA was expressed on mature lymphocytes (such as B cells, T cells, and Tregs), macrophages, and mature bone marrow-derived DCs [130]. Herpesvirus entry mediator (HVEM), a member of the tumor necrosis factor receptor superfamily (TNFRSF), was identified as the unique BTLA ligand in 2005 [131]. But BTLA was not the unique binding partner for HVEM, it competed with other two TNF family members, LIGHT and lymphotoxin-α, as well as IgSF member CD160 for binding to HVEM [132]. CD160 is another negative regulator of T cell while LIGHT is a costimulatory molecule [133, 134]. The ligation of BTLA with HVEM triggered the inhibition of T cell proliferation and cytokine production [131]. At present, there are no clinical trials opened for BTLA. But, in the past April, Junshi Biosciences announced that the world’s first anti-BTLA mAb, TAB004/JS004, have been approved for clinical trial by FDA [135].

A recent publication reported a new immune suppressor, sialic acid-binding immunoglobulin-like lectin 15 (Siglec-15) [136]. The team of Dr. Lieping Chen elegantly demonstrated the expression of Siglect-15 (mainly on cancer cells, macrophages, and myeloid cells) and the inhibitory role of Siglect-15 in regulation of T cell responses. More importantly, they revealed that both genetic ablation and antagonize antibody of Siglec-15 suppressed the growth of tumor in murine models [136]. Right now, a clinical trial lead by Chen’s group is recruiting to test the efficacy of NC318 (an anti-Siglec-15 mAb) in solid tumors (NCT03665285) [136, 137].

## Conclusion

The success of CTLA-4 or PD-1/PD-L1 blockade catalyzed the enthusiasm for a new class of antibody that block negative immune checkpoint regulators for cancer therapy. The past two decades have witnessed the significant progress in identifying alternative targets and developing novel specific agents in treating cancer. As we described above, considerable immune checkpoints have been explored with some been chosen as novel therapeutic targets by pharmaceutical companies. Although the number of clinical trials about these emerging immune modulators, such as anti-LAG-3 antibody and anti-TIM-3 antibody, registered on ClinialTrial.gov has grown exponentially, no drugs entered the clinic up to date. There are still some puzzles to be solved, like identification of ligand for VISTA and B7-H3 which may be the key to fully understand their therapeutic potential. In addition, while the results of monotherapy treatments are compelling, more attempts should be made to design rational combinations of immune-therapeutics that target non-redundant pathways to achieve synergistic effects in inhibiting tumor growth. We are still in the early stage of understanding these new immune systems with the aim that more candidates’ agents can transform to clinical fields and achieve even greater success than that initially observed with CTLA-4 or PD-1 blockade.

## Data Availability

Data sharing not applicable to this article as no datasets were generated or analyzed during the current study.

## Abbreviations

AEs: Adverse events
B7-H3: B7 homolog 3 protein
BTLA: B and T cell lymphocyte attenuator
Ceacam-1: Carcinoembryonic antigen cell adhesion molecule 1
CNS: Central nervous system
CTLA-4: Cytotoxic T lymphocyte-associated antigen-4
DC: Dendritic cells
FDA: United States Food and Drug Administration
HMGB1: High-mobility group protein B1
HVEM: Herpesvirus entry mediator
ICI: Immune checkpoint inhibitor
IgSF: Ig superfamily
IgV: Ig variable region
ITIM: Immune receptor tyrosine based inhibitory motif
ITSM: Immune receptor tyrosine-based switch motif
ITT: Immunoglobulin tail tyrosine
LAG-3: Lymphocyte activation gene-3
mAb: Monoclonal antibody
MBC: Metastatic breast cancer
MHC: Major histocompatibility complex
NK: Natural killer
NSCLC: Non-small cell lung cancer
ORR: Objective response rate
PD-1: Programmed cell death protein-1
PD-L1: Programmed cell death ligand 1
PR: Partial response
PtdSer: Phosphatidyl serine
RCC: Renal cell carcinoma
SHP1/2: Src homology 2 domain containing phosphatases 1/2
Siglec-15: Sialic acid-binding immunoglobulin-like lectin 15
SITC: Society for Immunotherapy of Cancer
TCR: T cell receptor
TIGIT: T cell immunoglobulin and ITIM domain
TIM-3: T cell immunoglobulin and mucin-domain containing-3
TME: Tumor microenvironment
Tregs: Regulatory T cells
VISTA: V-domain Ig suppressor of T cell activation
